# The Field of Science

**Published:** 1894-02-03

**Authors:** 


### THE FIELD OF SCIENCE.

A curious and audacious robbery has been perpetrated quite recently at the Laviboisière Hospital. What actually prompted his action we cannot tell, but suffice it to say a man called Jehan has made off with several corpses which were for the time being in the keeping of the authorities of the institution. Upon conviction Jehan has been condemned to an eight months' imprisonment.

Professor Uffelmann, of Rostock, gives us the results of his experiments regarding infection which is transmittable in correspondence through the medium of the post. One experiment in this direction was to infect a letter with cholera bacilli before placing it in the pillar-box. Upon the receipt of the letter 23½ hours after it was dispatched the bacilli were found to be yet alive. The Professor further discovered that there was little immunity of the risk thus run in cases of infection through the correspondence being carried on on post-cards; here the bacilli were also found to remain alive after a lapse of 24 hours.

Great interest is being shown in Russia in connection with the proposed International Medical Congress for 1896. The sum of 50,000 roubles has been voted towards the expenses of the project. In all probability Moscow will be decided on as the meeting-place since the Government particularly favours the selection of this city for the projected Congress.

In illustration of the universal hold that "preventive measures" have taken upon literate and illiterate minds alike, we give the following quain example, which appeared in an American country newspaper. It is all the more deserving of notice, as being incorporated in the report of the local Board of Health: "Every person who is confined in a house, owing to sickness and contagious diseases, should at all times be thoroughly covered with disinfectants both externally and internally, to ensure safety to themselves and to others, as in my mind a spread of the same is caused by carelessness on the part of people who know it all, and cannot be told by persons of experience."

